# Giant virilising adrenal cortical carcinoma

**DOI:** 10.4322/acr.2021.259

**Published:** 2021-05-06

**Authors:** Shruti Dogra, Arvind Ahuja, Minakshi Bhardwaj, Rohan Sardana, Hemant Goel

**Affiliations:** 1 Atal Bihari Vajpayee Institute of Medical Sciences, Dr Ram Manohar Lohia Hospital, New Delhi, India.

**Keywords:** Adrenocortical carcinoma, Adrenal Gland, Functional, Giant, Largest

## Abstract

Androgen secreting adrenocortical carcinoma (ACC) is a very rare disease with a poor prognosis. Approximately 80% of tumors are functional, most commonly secreting glucocorticoids. We herewith report a case of a huge functional ACC of the right adrenal gland in a 33-year-old female who presented with complaints of hirsutism, amenorrhea and an abdominal lump. On abdominal examination a large lump was palpable in the right hypochondrium reaching up to the umbilicus. Contrast-enhance computed tomography (CECT) revealed a mass in the right suprarenal region. The tumor measured 29 cm × 20 cm × 12 cm and weighed 7.8 kg, the largest reported case of ACC in the world to the best of our knowledge.

## INTRODUCTION

Adrenocortical carcinomas (ACC) are rare tumors with an incidence of 0.5-2 cases per one million population per year and constitute 0.05-0.2% of all malignancies.^1^ There is a bimodal age distribution with peaks in the first decade and in the fifth decade of life.^2^ ACCs develop somewhat more common in women than men in most large series and measure about 10 to 12 cm. Conventional ACCs are typically large tumors and, as a rule, weigh more than 100gms in adults. Mostly these tumors weigh around 750gms. There is considerable variability in the reported functional status of adrenocortical carcinomas. Approximately 80% of tumors are functional, most commonly secreting glucocorticoids (45%), glucocorticoids and androgenic steroids (45%), and androgenic steroids alone (10%). Rarely, mineralocorticoid production may be present.^3^ Functioning ACC presents earlier with hormonal manifestations such as virilization, feminization or Cushing’s syndrome. Nonfunctioning tumors pose a diagnostic challenge because they are diagnosed incidentally due to a mass effect or metastatic disease.^4^

We report a case of the largest androgen-producing, non-metastasizing ACC mass till date. The patient underwent right radical nephrectomy with in toto excision of the tumor. On gross examination the tumor measured 29×20×12 cm. On microscopy and by immunohistochemistry (IHC) a diagnosis of ACC was confirmed. After performing an extensive google search of all the articles published on ACCs, to date, we report this case as the largest ACC in size in the world.

## CASE REPORT

**Figure 1:**
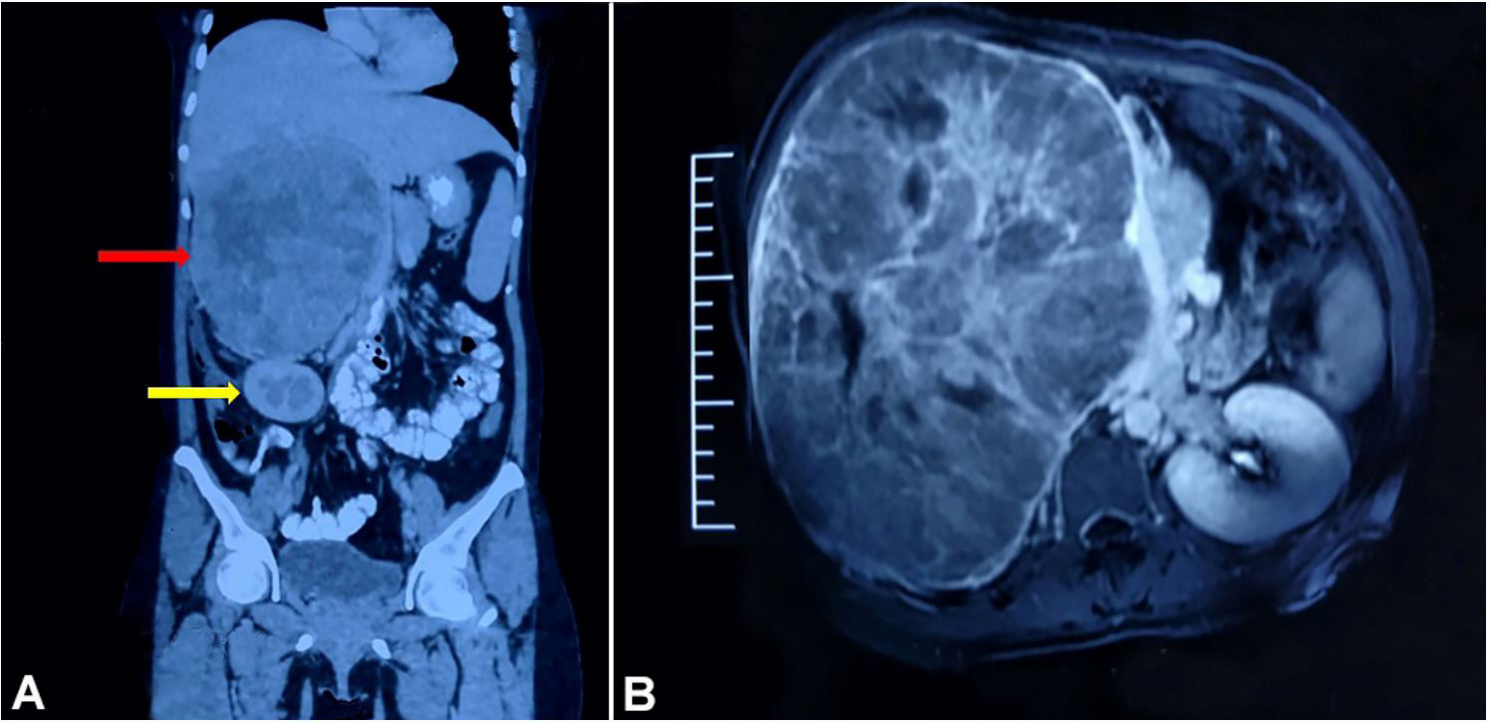
**A** and **B** – CECT showing a large solid hypodense lesion measuring 24×20×16 cm, in the right adrenal area displacing the portal vein, pancreas and IVC to the left side (red arrow). Mass is also compressing right kidney (yellow arrow).

A 33-year-old female presented with cessation of menses along with hair growth on face and body over the last 2 years. There was no history of hypertension, diabetes, tuberculosis or any other chronic disorder. She was on oral contraceptive pills for 5 months and had a 5-year-old child at the time of the first hospital visit. On examination, an abdominal lump was found in the right hypochondrium reaching up to the umbilicus and midline measuring ~ 20×20×15 cm. It was soft in consistency, non-tender with no ascites. There was no audible bruit or any other palpable lump. Her average blood pressure was 130/80 mmHg on 24-hour ambulatory blood pressure monitoring. Laboratory investigations revealed raised serum cortisol~ 22.53 µg/dl (Reference range [RR]; 4.30-22.40µg/dl) and serum total testosterone~337.76 ng/dl (RR; 14-76ng/dl). Blood sugar levels, renal function tests, liver function tests, serum electrolytes, hormone profile including serum aldosterone, and 24-hour urine metanephrines levels were within normal limits. A triphasic abdominal and pelvic contrast-enhanced computed tomography (CECT) demonstrated a large solid hypodense mass measuring 24×20×16 cm, with areas of necrosis and increased arterial blood supply (mostly direct branches of aorta) in the right adrenal topography. The right adrenal was not seen separately. The mass had displaced the portal veins, pancreas, inferior vena cava to the left side, and compressed the right kidney (Figure 1AB).

**Figure 2:**
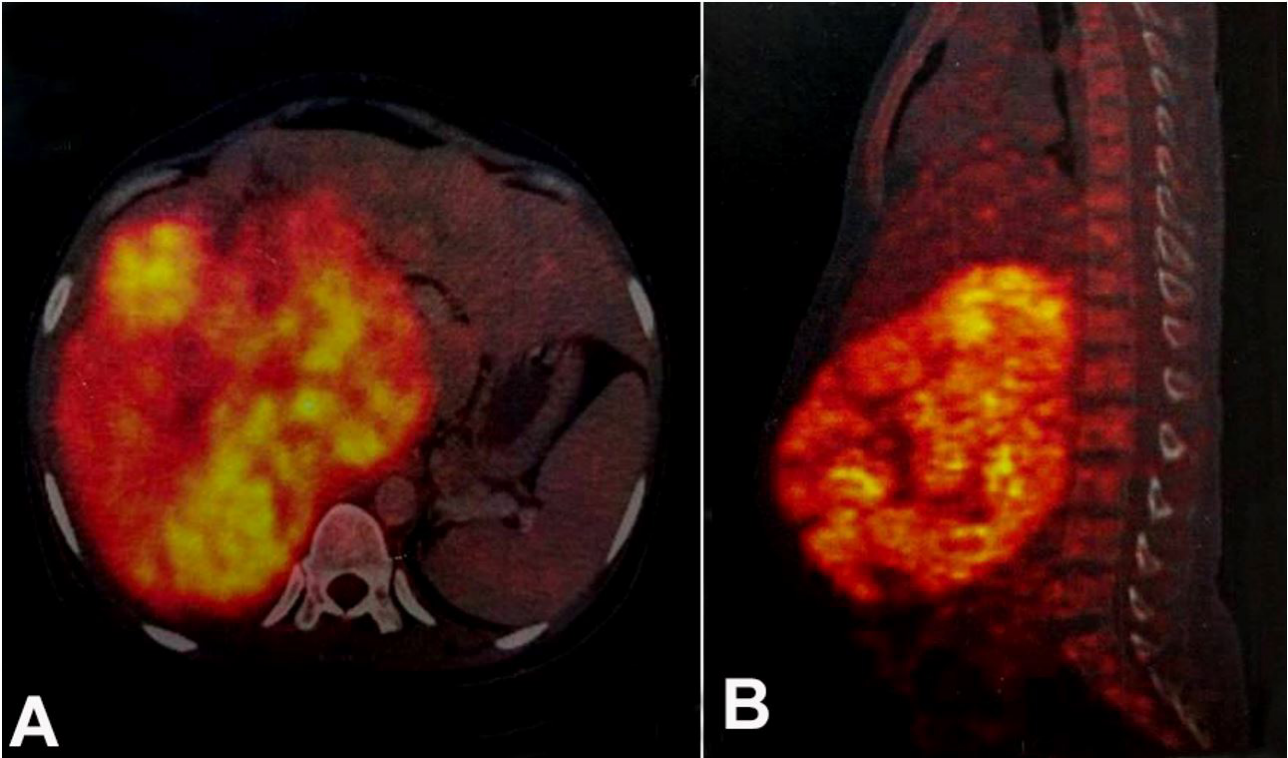
**A** and **B** – PET-CT showing large FDG avid heterogeneously enhancing lobulated soft tissue density mass lesion in the right suprarenal region. Right adrenal gland was not separately visualized.

Positron emission tomography-computed tomography (PET/CT) was performed for metastatic workup showed a large FDG avid heterogeneously enhancing lobulated soft tissue density mass lesion in the right suprarenal region (Figure 2AB). Right adrenal gland was not separately visualized and no other abnormal hypermetabolic focus was seen elsewhere in the body. 

Tumor markers CA 15.3, CA125, CA 19.9 and AFP were not elevated. An image-guided biopsy was performed in some other institute where it was reported as consistent with an epithelial neoplasm with oncocytic, clear phenotype, and angular nesting growth pattern. Tumor cells were positive for Melan A, Calretinin and Vimentin suggesting an adrenal origin. The Ki 67 was 5-6%. The patient underwent radical nephrectomy with in toto excision of the tumor. Per operatively a large adrenal mass (right) reaching up to the pelvis crossing midline, and touching but not invading the lower border of liver and inferior vena cava and encroaching right kidney was found and sent for histopathological examination.

**Figure 3:**
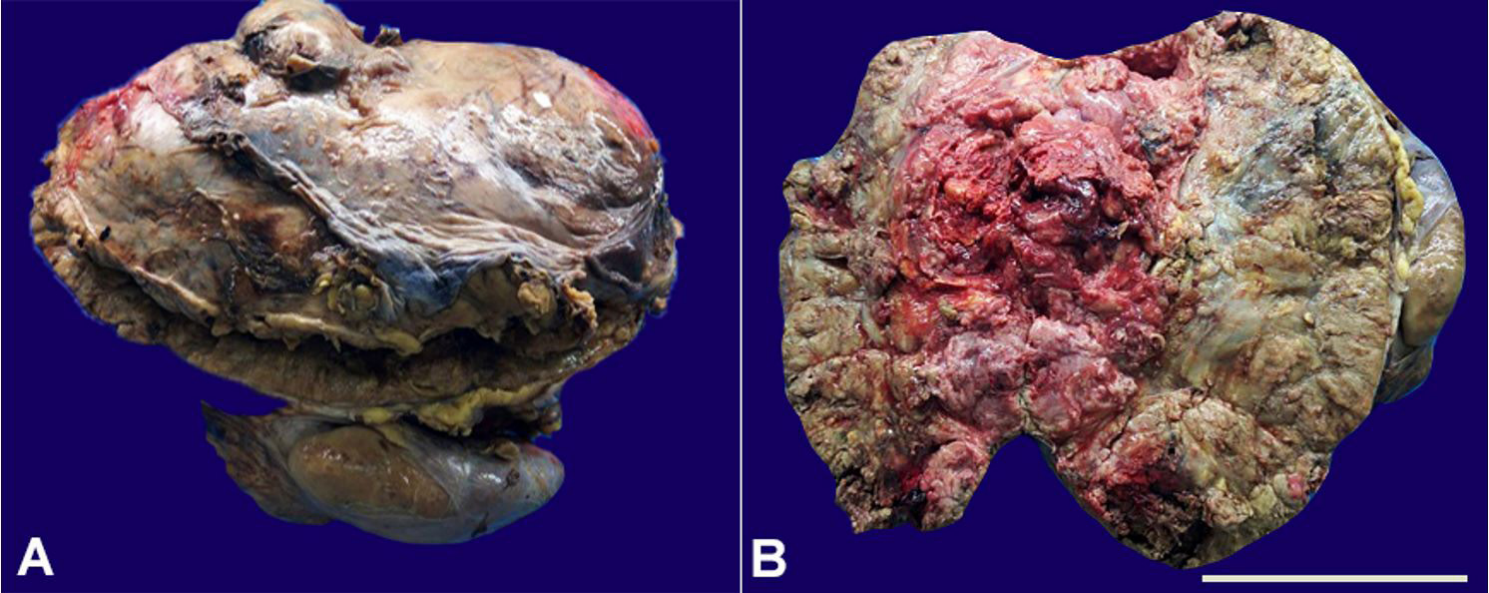
**A** and **B** – Gross of giant adrenal mass measuring 29×20×12 cm with cut surface showing a variegated appearance and large areas of necrosis. (Scale bar = 10 cm)

Grossly the large adrenal mass with the intact capsule measured 29×20×12 cm and weighed 7.8kg (Figure 3A). The cut surface showed a variegated appearance with large areas of necrosis(Figure 3B). The kidney measured 12×7×3 cm, and its cut section showed normal renal parenchyma with maintained cortico-medullary junction.

**Figure 4:**
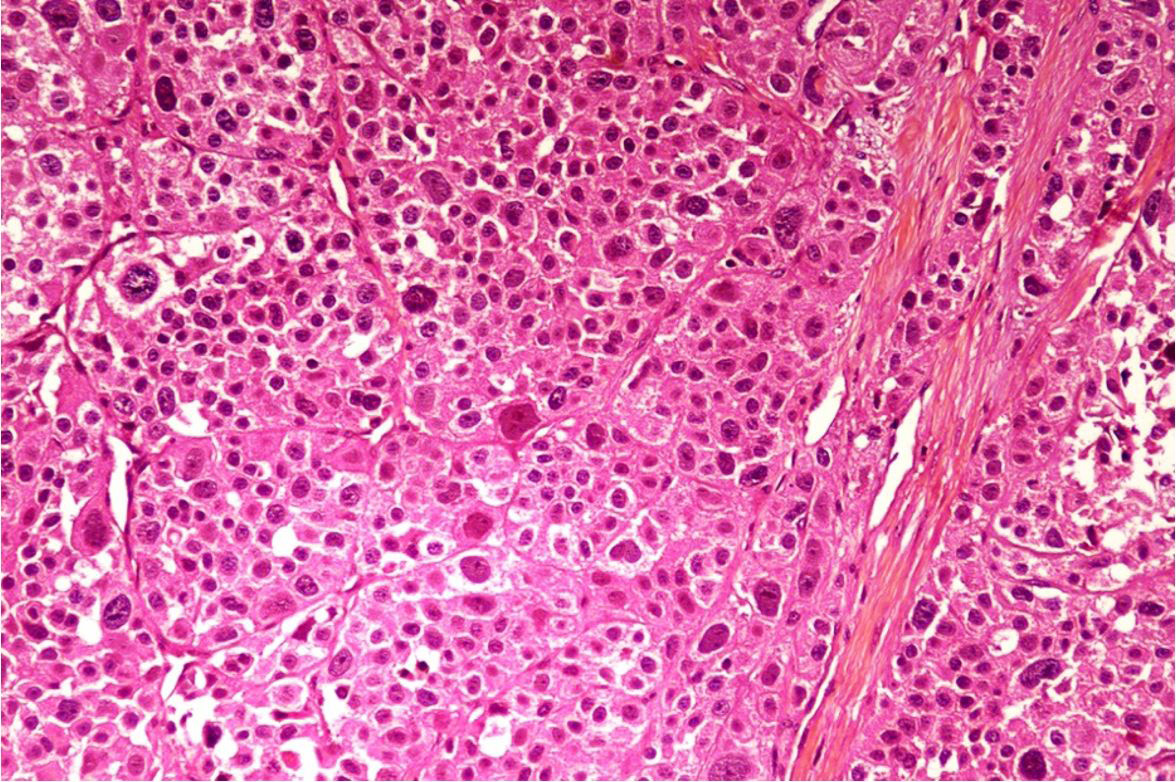
Photomicrograph showing highly cellular tumor composed of cells having markedly pleomorphic round to oval nuclei, coarse granular chromatin, prominent nucleoli and moderate amount of eosinophilic to clear cytoplasm. Many bizarre cells and brisk mitotic activity is also present (H&E; 40x).

Microscopic sections from the adrenal mass showed a highly cellular tumor composed of cells arranged in sheets and nests having markedly pleomorphic round to oval nuclei, coarse granular chromatin, prominent nucleoli and moderate amount of eosinophilic to clear cytoplasm. Many bizarre cells and brisk mitotic activity was noted along with large areas of necrosis (Figure 4). Capsular and vascular invasion were not seen. Sections from periadrenal fat, renal cortex, medulla and hilum were free from tumor.

**Figure 5:**
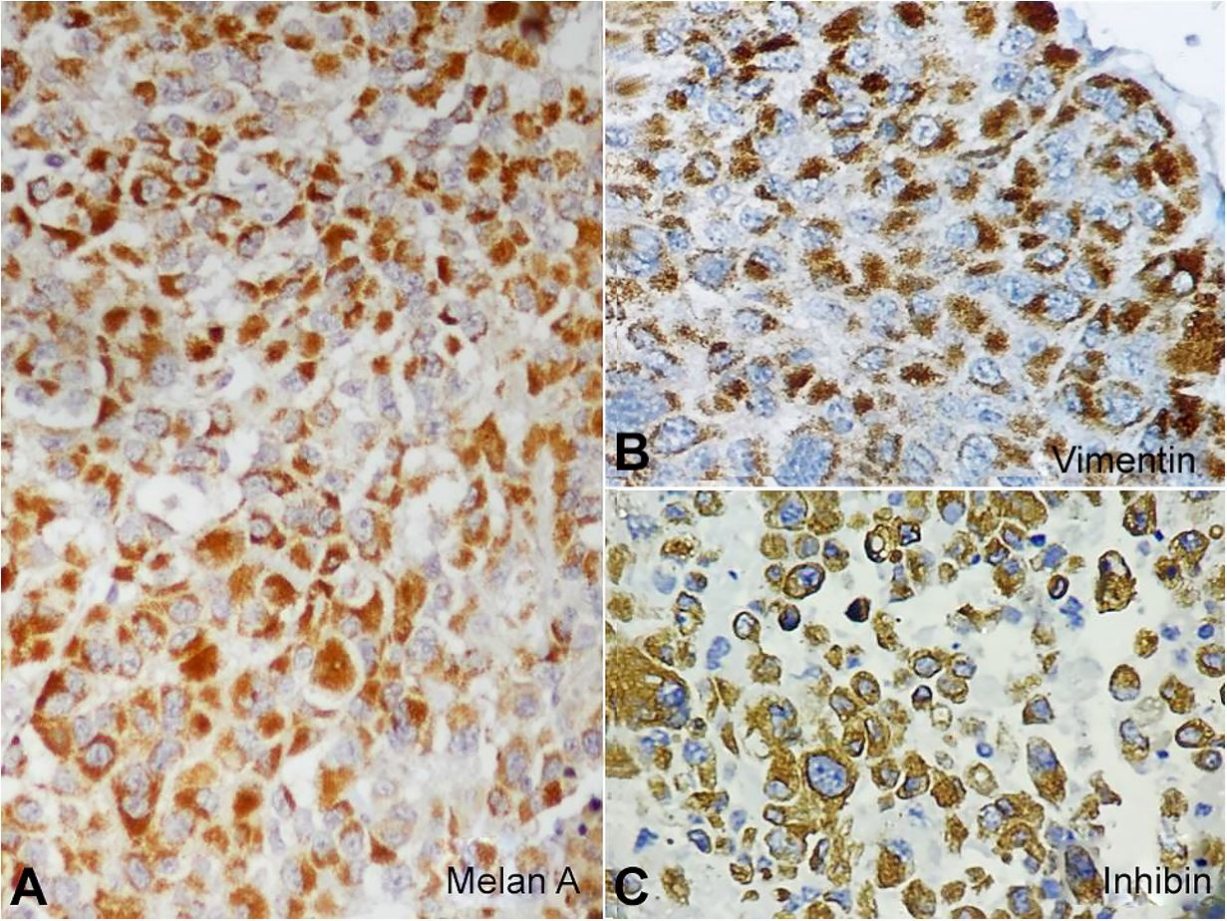
**A** and **B** – Tumor cells showing strong cytoplasmic positivity for Melan A & Vimentin (Figure 5A-B); **C** – Focal weak positivity for Inhibin (IHC; 40x) (Figure 5C).

On the immunohistochemical (IHC) study, the tumor cells were strongly positive for Melan A and Vimentin (Figure 5AB). Inhibin was weak focal positive (Figure 5C). The tumor was negative for Chromogranin, Synaptophysin, EMA, CK7 and CK20.

A final diagnosis of adrenocortical carcinoma, modified Weiss score 4, pathological stage T2N0M0, Stage II was made. Therefore, the patient was advised 6 monthly follow up PET scans with no adjuvant chemotherapy. On two year follow up the patient had no recurrence and is doing well with a normal hormonal status.

## DISCUSSION

Primary adrenocortical tumors are large tumors usually measuring more than 5 cm at presentation. The larger the tumor, the greater is the chance of being malignant. The present case was malignant and measured 29×20×12 cm. Didolkar et al.^5^ have studied the natural history of a large series of patients with adrenocortical carcinoma in which the mean duration of symptoms in patients with or without hormonal manifestations was 6 months. Fifty-two percent of patients had distant metastases at the time of diagnosis, 41% had locally advanced disease, and 7% had tumor confined to the adrenal gland. In our case the patient was symptomatic for 2 years; however, the tumor was confined to the adrenal at the time of diagnosis. The overall median survival was 14 months, and the 5-year survival rate was 24%.^5^ The median survival of patients with functional tumors was somewhat longer than that of patients with nonfunctional tumors. Our case was of a functional tumor with the patient doing well after two years of surgery.

The most common sites of metastasis are the lungs, followed by retroperitoneal lymph nodes, liver, and bone. Because they are large, the organ of origin is often difficult to determine. Therefore, the diagnosis on biopsy is difficult based on morphology alone and a panel of immunohistochemistry is required to rule out other differentials. In these instances, a battery of immunostains can provide evidence of adrenocortical differentiation which include α-inhibin, calretinin, vimentin, synaptophysin, Melan A and steroidogenic factor 1 (SF1). Metastasis to the adrenal is the other possibility. The most common tumors metastasizing to the adrenals are lung carcinoma, renal cell carcinoma, breast carcinoma, and melanoma. However, in the setting of metastatic carcinoma, bilateral adrenal masses are more likely.^6^ However, in surgical specimens a thorough examination of the tumor capsule, looking for capsular and vascular invasion accompanied by an extensive sampling of the tumor, to ensure that a high-grade component is not missed, should be done. The main differential diagnoses are adrenocortical adenoma, pheochromocytoma, renal cell carcinoma and hepatocellular carcinoma.

After extensive review of literature, we report a case of the largest adrenocortical carcinoma. A brief review of the largest reported cases has been depicted in Table 1. Other cases reported were all less than 20 cm in size.^4^^-^^10^ The initial classification of ACC took into account the size of the tumor as determinant to the diagnosis of malignant behavior. However, currently, along with tumor size, histologic criteria evolved to incorporate a variety of histological, and immunohistochemical parameters. The Weiss system^11^ and its modifications have gained the most acceptance in clinical practice as the diagnostic criteria for ACC. Modified Weiss criteria include the following criteria: (i) mitotic rate > 5 per 50 high power fields, (ii) cytoplasm (clear cells comprising 25% or less of the tumor), (iii) abnormal mitoses, (iv) necrosis and (v) capsular invasion. To calculate each criterion is scored 0 when absent and 1 when present: 2x mitotic rate criterion + 2x clear cytoplasm criterion + abnormal mitoses + necrosis + capsular invasion (score of 3 or more suggests malignancy). The modified Weiss score of our case was 4.

**Table 1:** Review of previous reported cases of functional large size Adrenocortical carcinomas (ACC) and their management

**Case**	**Patient Age/Sex**	**Presentation**	**Type of tumor**	**Tumor size cm**	**Tumor weight (g)**	**Management**
Kunieda et al.^7^	52Y/M	Weight loss & Abdominal discomfort	Functional ACC	29×19×10	4700	Tumor extirpation with partial liver resection
Straka et al.^8^	40Y/M	Respiratory distress	Functional ACC	26×16×13	2372	In toto excision of tumor
Uruc et al.^9^	48Y/F	Abdominal pain & Flushing	Functional ACC	23×18×16	1300	En bloc removal of tumor
Present case	33Y/F	Amenorrhea & Hirsutism	Functional ACC	29×20×12	7800	In toto excision of tumor with radical nephrectomy

Currently, complete tumor resection is the only curative approach to ACC. Adjuvant chemotherapy has a role in decreasing the chance of recurrence and increase survival.^12^ Also large tumor size (diameter > 12 cm) has been associated with poor survival after complete resection.^2^ Although our case is the largest case of ACC , the patient is doing well at 2 year follow up. Postoperative surveillance should be performed at every 3 months for the first 2 years and then every six months for 5 years to look for recurrence or metastasis by FDG-PET/CT scan. The prognosis is highly stage-dependent and over the years several staging systems have been proposed. Currently, the ENSAT (European Network for the Study of Adrenal Tumors) classification is commonly used. Five-year survival for patients with disease confined to adrenal gland is size-dependent, varies from 61% to 82%; those with distant metastasis at diagnosis have a five-year survival of only 18%.^13^

## CONCLUSION

ACC is a rare malignancy with a size dependent prognosis. This case has been reported because of the gigantic size yet non metastasizing nature of the tumor. The case was managed by complete surgical removal and had an excellent prognosis despite the large size, as the tumor did not invade the nearby organs and was removed in toto. To the best of our knowledge, this is the largest ACC reported till date.
